# The Influence of Formulation and Manufacturing Process Parameters on the Characteristics of Lyophilized Orally Disintegrating Tablets

**DOI:** 10.3390/pharmaceutics3030440

**Published:** 2011-07-20

**Authors:** Rhys J. Jones, Ali Rajabi-Siahboomi, Marina Levina, Yvonne Perrie, Afzal R. Mohammed

**Affiliations:** 1 Aston Pharmacy School, Aston University, Birmingham, B4 7ET, UK; 2 Colorcon, Inc., Harleysville, PA 19438, USA; 3 Colorcon Limited, Flagship House, Victory Way, Crossways, Dartford, DA2 6QD, UK

**Keywords:** freeze-drying, orally disintegrating tablets, gelatin, pH, ionic strength, milling

## Abstract

Gelatin is a principal excipient used as a binder in the formulation of lyophilized orally disintegrating tablets. The current study focuses on exploiting the physicochemical properties of gelatin by varying formulation parameters to determine their influence on orally disintegrating tablet (ODT) characteristics. Process parameters, namely pH and ionic strength of the formulations, and ball milling were investigated to observe their effects on excipient characteristics and tablet formation. The properties and characteristics of the formulations and tablets which were investigated included: glass transition temperature, wettability, porosity, mechanical properties, disintegration time, morphology of the internal structure of the freeze-dried tablets, and drug dissolution. The results from the pH study revealed that adjusting the pH of the formulation away from the isoelectric point of gelatin, resulted in an improvement in tablet disintegration time possibly due to increase in gelatin swelling resulting in greater tablet porosity. The results from the ionic strength study revealed that the inclusion of sodium chloride influenced tablet porosity, tablet morphology and the glass transition temperature of the formulations. Data from the milling study showed that milling the excipients influenced formulation characteristics, namely wettability and powder porosity. The study concludes that alterations of simple parameters such as pH and salt concentration have a significant influence on formulation of ODT.

## 1. Introduction

Orally disintegrating tablets (ODTs), which are also referred to as orodispersible and fast disintegrating tablets, are tablets which when placed in the mouth, disperse/disintegrate rapidly before being swallowed, due to the action of saliva [1]. The Food and Drug Administration recommends that ODTs be considered as solid oral preparations that disintegrate rapidly in the oral cavity, with an *in vitro* disintegration time of approximately 30 seconds or less, when based on the United States Pharmacopeia disintegration test method or alternative [2]. This form of solid dosage form is therefore highly applicable for groups of the population who commonly have difficulty in swallowing conventional solid dosage forms (e.g. conventional tablets and capsules), such as pediatric and geriatric patients [3].

A number of technologies have been used to manufacture ODTs including freeze-drying (lyophilization), molding and conventional compression methods [4]. More recently new technologies such as tablet loading [5], compression of pulverized components [6] and sublimation [7] have also been reported.

However, ODTs manufactured using freeze-drying have been the most successful commercially. Tablets manufactured using this technology, generally exhibit rapid disintegration and dissolution due to their highly porous nature, which allows penetration of saliva into the matrix of the tablets, resulting in disintegration. The freeze-drying process involves the transition of water from liquid to solid during freezing, and then solid to vapor during sublimation [8]. A particular advantage of freeze-drying is that the solution is frozen such that the final dry product is a network of solid occupying the same volume as the original solution, resulting in a light and porous product which is readily soluble [8].

Gelatin and mannitol are both excipients which are used in the formulation of freeze-dried ODTs [9]. These materials are responsible for forming the highly porous matrix structure of the dosage form. Gelatin, a protein, which acts as a glassy amorphous compound, provides structural strength, whilst mannitol (a sugar alcohol) provides crystallinity, hardness and elegance [9]. Water is used as a manufacturing process media, which induces the porous structure upon sublimation during the freeze-drying stage [9].

Interestingly, studies investigating molecular variations in gelatin configuration have shown that various factors such as pH and salt concentration influence its packing and solubility. A pH-swelling curve for lime processed gelatin (type B) has been reported [10]. The results showed that adjusting the pH away from the isoelectric point resulted in a significant swelling of the material. Subsequently, the swelling properties of gelatin could potentially be utilized to increase the porosity of the freeze-dried tablet matrix, and could lead to a reduction in disintegration time. Another study investigating the solubility of gelatin has shown that it exhibits its lowest solubility at pH 5 (isoelectric point), with improvements in solubility above the isoelectric point [11,12]. Other factors that have shown to influence gelatin swelling and solubility include the addition of neutral salts and variations in ionic strength of the formulation [12,13]. Despite the availability of literature on gelatin behavior, there has been no work reported in exploiting gelatin properties under different conditions in the formulation of ODTs.

Besides varying parameters such as pH and ionic strength that will potentially influence the physicochemical properties of gelatin, another factor which has received very little attention is particle size reduction. Ball milling is a widely used technique to reduce particle size [14] and has been shown to influence the transition of materials from crystalline to amorphous form [15,16]; to change the performance of a variety of dosage forms by improving their solubility [17], dissolution [18], and bioavailability [19].

The aim of the current study was to exploit the various process parameters such as adjustment of pH, ionic strength of the formulation and ball milling to study their influence on tablet properties with the aim of reducing disintegration time without compromising tablet hardness. The formulated tablets used in this study consisted of 9% w/w gelatin and 30% w/w (of the dried tablet weight) mannitol, as excipients. The choice of formulation was influenced by the preliminary results which showed that the above combination exhibited high tablet hardness and long disintegration time (around two minutes).

## 2. Experimental Section

### 2.1. Materials

Gelatin (type B, 60 and 75 bloom) and mannitol were supplied by Sigma-Aldrich Chemicals (Poole, UK). HPLC grade methanol was supplied by Fisher Scientific (Loughborough, UK). Ibuprofen, sodium chloride and sodium hydroxide were supplied by Sigma-Aldrich Chemicals (Poole, UK). Hydrochloric acid was supplied by Fisher Scientific (Loughborough, UK). All chemicals were used without further purification.

### 2.2. Methods

#### 2.2.1. Preparation of Freeze-Dried Tablets

Gelatin was dissolved in double-distilled water at about 40 °C, followed by the addition of mannitol to form a solution. 1.5 g of the resulting solution was dosed into a tablet mould, frozen at −70 °C for a minimum of sixty minutes and freeze-dried (ADVANTAGE, Freeze-Dryer, VIRTIS) according to the following regime; primary drying for forty eight hours at a shelf temperature of −40 °C, secondary drying for ten hours at a shelf temperature of 20 °C and vacuum pressure of 50mTorr. A minimum of ten tablets were prepared for each formulation.

#### 2.2.2. Ball Milling

Mixtures of gelatin and mannitol were milled using a planetary micro mill (FRITSCH Pulverisette 7, Germany), with 45 mL agate grinding bowls and 10 mm diameter agate balls at room temperature. The milling process was performed under various conditions, as shown in Table 1, in order to investigate the effect of the milling parameters, *i.e.*, milling time, rotation speed, and ball: powder weight ratio, on the; wettability, porosity of the milled samples and glass transition of the formulations in their frozen state prior to freeze-drying. Ultimately the effect of milling on the properties of the freeze-dried tablets, namely; disintegration time, porosity, hardness and fracturability were investigated. The weight of the gelatin-mannitol mixture was 3 g for all of the milling conditions. The milling parameters were determined using MODDE factorial-experimental design software.

#### 2.2.3. Differential Scanning Calorimetry (DSC)

DSC (Pyris Diamond DSC and Intercooler 2P: Perkin Elmer, Wellessey, USA) was used to determine the glass transition temperature (*T*_g_) of the formulations in their frozen state (before freeze-drying). 10–15 mg of the liquid samples were loaded into aluminum pans, cooled to −65 °C at a rate of 5 °C/min with a nitrogen purge of 20 mL/min, an empty aluminum pan was used as a reference for all measurements.

The resulting thermograms were analyzed by Pyris manager software. *T*_g_ values were determined from the intersection of relative tangents to the baseline. Three samples/measurements were taken for each formulation, and the mean values ± standard error were reported.

#### 2.2.4. Wettability Analysis

The wettability of the milled and non-milled (control formulation) samples were analyzed using the Wilhelmy method, to determine their contact angle. A Camtel^©^ (Hertfordshire, UK) QCT-100 surface tensiometer was used to determine the contact angle and subsequent wettability of the samples.

Glass slides measuring 24 × 24 mm in size were covered with 12 × 24 mm double-sided adhesive tape. The glass slides were then placed in the various formulations, to coat them. Excess formulation was removed by gentle tapping to ensure a uniform coat. Glass slides were then securely attached to the microbalance of the apparatus, and during wettability analysis, a glass beaker containing the test liquid (80 mL of double distilled water) was raised and lowered at a rate of 0.200 mm/s, to immerse the glass slides. During this period, contact angles were determined automatically at regular intervals.

Each formulation was analyzed in triplicate for their greatest contact angle, and mean ± standard error is reported. The test liquid was replaced for analyzing each formulation.

A linear relationship between wetting time (wettability) and disintegration time of rapidly disintegrating tablets has been reported [20]. Therefore, analysis of the wettability of excipients is a useful tool in understanding the performance (disintegration time) of fast disintegrating tablets.

#### 2.2.5. Powder Porosity Analysis

The porosity of the milled and non-milled (control formulation) samples were measured using helium pycnometry (MULTIPYCNOMETER, Quantachrome Instruments, Hampshire, UK). 1 mL of sample was placed in a suitably sized sample cup and subjected to helium pycnometry, to determine the true density of the sample. The true density value was then used in the following equation (Equation 1) to determine the porosity of the sample:

Equation 1 Porosity=(1−bulk density/true density)×100%

Bulk density was determined by considering, the mass and volume of the sample. Three porosity measurements were taken for each formulation, and the mean ± standard error is reported. The porosity of the samples was expressed as a percentage.

#### 2.2.6. Mechanical Properties of the Tablets

The mechanical properties of the tablets (hardness and fracturability) were investigated with a texture analyzer (QTS 25: Brookfield, Essex, UK) equipped with a 25 kg load cell. The instrument was calibrated by standard weights of 500 g and 5 kg. The tablets were placed in a holder with a cylindrical hole. The hardness was taken as the peak force after 1mm penetration of a 5 mm diameter probe at a rate of 6 mm/min. Three measurements were taken for both hardness and fracturability, for each formulation, and the mean ± standard error is reported.

#### 2.2.7. Disintegration Time of the Tablets

The disintegration time of the tablets was determined using a USP disintegration tester (Erweka, ZT3). 800 mL of double distilled water, which was kept at 37 ± 2 °C, was used as the medium and the basket was raised and lowered at a fixed rate of 30 per minute. Three tablets were evaluated from each formulation, and the mean values ± standard error is reported.

#### 2.2.8. Tablet Porosity

The porosity of the tablets was measured using helium pycnometry (MULTIPYCNOMETER, Quantachrome Instruments, Hampshire, UK). Two freeze-dried tablets were placed in a suitably sized sample cup and subjected to helium pycnometry, to determine the true density of the tablets. The true density value was then used in the equation, as reported above (Equation 1), to determine the porosity of the tablets.

Bulk density was determined by considering tablet weight, diameter and thickness. The diameter and thickness of the tablets were determined using a screw gauge (LINEAR Farnell). Three porosity measurements were taken for each formulation, and the mean values ± standard error is reported. The porosity of the tablets is expressed as a percentage.

#### 2.2.9. Statistical Analysis and Factorial-Experimental Design

The effect of milling on the performance and properties of freeze-dried tablets was compared to those of the control (non-milled formulation); using one-way analysis of variance (ANOVA) with the Dunnett multiple comparisons test. The significant effect of treatment/level of statistical significance was judged as being *p* < 0.05, with a confidence limit of 95%. This statistical analysis test was also used for the ionic strength study.

In terms of factorial-experimental design, the milling factors consisted of; milling time (ranging from 15–60 minutes), rotation speed (ranging from 100–400 rpm) and ball:powder weight ratio (ranging from 5–15). Eleven formulations were proposed, which underwent different milling conditions, according to the three factors, as shown in Table 1.

The responses measured included excipient properties; wettability, powder porosity and glass transition (of the formulations in their frozen state, prior to freeze-drying). The responses measured also included tablet properties; disintegration time, porosity, hardness and fracturability.

Statistical analysis of the dissolution of ibuprofen from tablets prepared from both non-milled and milled excipients was performed using the unpaired t-test with Welch correction. This statistical analysis test was also used for the pH study. Ibuprofen was used as a model active pharmaceutical ingredient (API), as it is a readily available API. Also, the majority of API's used in freeze-dried ODTs are insoluble or poorly soluble in water, and as ibuprofen is poorly soluble in water, it was deemed as a suitable model API to use in the dissolution study.

#### 2.2.10. Morphological Examination

The inner structural morphology and pore size of the freeze-dried tablets were examined by scanning electron microscopy (SEM, STEREOSCAN 90, Cambridge Instrument). Thin horizontal and vertical samples of the tablets were prepared by cutting them with a surgical blade. The samples were placed onto double-sided adhesive strips on aluminum stubs and coated with a thin layer of gold using a sputter coater (Polaron SC500, Polaron Equipment, Watford, UK) at 20 mA for three minutes (this was performed twice for each set of samples) and then examined by the SEM. The acceleration voltage (kV) and the magnification can be seen on each micrograph. The pore size of the freeze-dried tablets was measured by using the scale which was visible on each micrograph. The average pore size was measured by measuring the pore diameter of around 10 randomly selected individual pores, from these values the average pore diameter was calculated.

#### 2.2.11. Dissolution Study and HPLC Conditions

The dissolution rate of ibuprofen from the two formulations (milled and non-milled tablet excipients), was examined using a Caleva 8ST dissolution bath. The two formulations were analyzed in triplicate. The dissolution system employed was USP dissolution apparatus 2 (paddle at 50 rpm rotation speed), for a test time of 60 minutes. The dissolution medium consisted of 900 mL of pH 7.2 phosphate buffer at 37 °C. Dissolution samples were filtered through a 0.45 μm Nylon syringe filter, to remove undissolved ibuprofen. 10 μL of the samples were analyzed by HPLC (Dionex), with UV detection performed at 230 nm, on a Thermo Scientific Hypersil Gold C18 250 × 4.6 mm 5 micron column, with methanol:water (80:20) as the mobile phase and a flow rate of 1.00 mL/min.

## 3. Results and Discussion

### 3.1. Influence of Changes in pH on Tablet Formation

The first phase of the study investigated the influence of changes in pH of formulation solution comprising of gelatin and mannitol. Three pH values of 3, 5 and 8 were chosen to determine the effect of pH variation below, at and above the isoelectric point of gelatin. The results showed that tablets prepared from solutions with pH adjusted to 3 resulted in severely denatured/degraded tablets when compared to formulations prepared at pH 5 and 8.

The instability of the formulation at pH 3, which included the detection of no *T*_g_, can be attributed to various reasons including acid hydrolysis of gelatin, maximum stability of gelatin at pH values between 5 and 8 and incompatibility of mannitol in strongly acidic solutions [21-23]. The lack of intact tablet formation resulted in no further characterization of formulations prepared at pH 3.

#### 3.1.1. Glass Transition and Tablet Mechanical Properties

DSC analysis of the formulation adjusted to pH 8, in its frozen state (prior to freeze-drying), indicated that this formulation exhibited a mean onset *T*_g_ of −29.1 ± 0.4 °C, whilst the control formulation (pH 5) exhibited a mean onset *T*_g_ of −29.8 ± 0.5 °C. The results have shown that pH adjustment does not have any plasticization effect or reduced the physical stability of the formulations in their frozen state (prior to freeze-drying).

Measurement of hardness of the resultant tablets prepared upon pH adjustment to 8 indicated a mean hardness of 60.0 ± 1.7 N, compared to 52.4 ± 8.8 N, the mean hardness of the tablets prepared at pH 5. Adjustment of the pH to 8 did not significantly vary the hardness when compared to pH 5 (*p* > 0.05). Similar results were obtained for fracturability studies.

#### 3.1.2. Tablet Porosity, SEM and Disintegration Time Analysis

**Figure 1.**
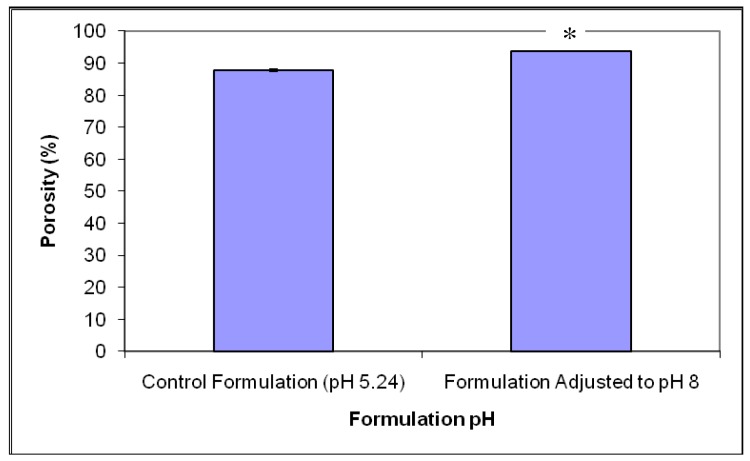
The effect of formulation pH on the porosity of the freeze-dried tablets (mean ± S.E., n = 3) (* statistically different).

Porosity analysis of the formulation adjusted to pH 8, as shown in Figure 1, indicated that there were significant differences between tablet porosity upon adjustment of pH. The mean porosity value of formulations prepared at pH 8 was 93.7 ± 0.1%, whilst the formulations prepared at pH 5 exhibited a mean porosity value of 87.7 ± 0.2% (*p* < 0.0001). The differences in porosity upon pH adjustment of the formulation can possibly be due to differences in swelling properties of gelatin upon change in pH. Previous studies have shown that increasing the pH above isoelectric point of gelatin resulted in greater swelling of strands due to variations in molecular chains of gelatin molecules thereby resulting in larger pore sizes within the tablet matrix [24,25].

**Figure 2.**
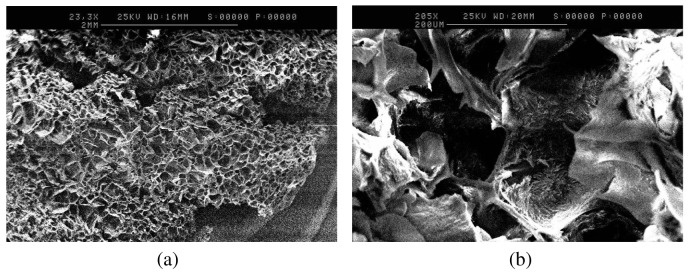
(**a**) SEM image of the tablet matrix of the formulation adjusted to pH 8. Horizontal Sample. Low Magnification, ×23; (**b**) SEM image of the tablet matrix of the formulation adjusted to pH 8. Horizontal Sample. High Magnification, ×205.

**Figure 3.**
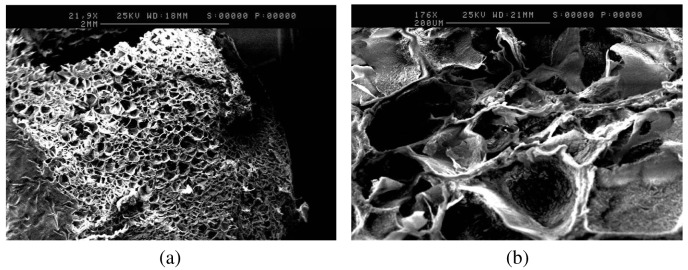
(**a**) SEM image of the tablet matrix of the control formulation. Horizontal Sample. Low Magnification, ×22; (**b**) SEM image of the tablet matrix of the control formulation. Horizontal Sample. High Magnification, ×176.

To further study the differences in porosity, anatomical studies using scanning electron microscopy were carried out. SEM analysis of the formulation adjusted to pH 8, as shown in Figure 2a,b, further supports the swelling behavior of gelatin upon pH variations. The 2-dimensional porous structure of the freeze-dried tablets of the formulation adjusted to pH 8, appeared to exhibit greater average pore diameter (100–140 μm) and thinner average pore wall thickness (20 μm) (Figure 2a,b), compared to the freeze-dried tablets of the control formulation (average pore diameter of 90 μm, and average pore wall thickness of 40 μm), as shown in Figures 3a and 3b. This observation can be attributed to the swelling behavior of gelatin at pH 8 [10] that results in the formation of larger pores with thinner walls.

**Figure 4.**
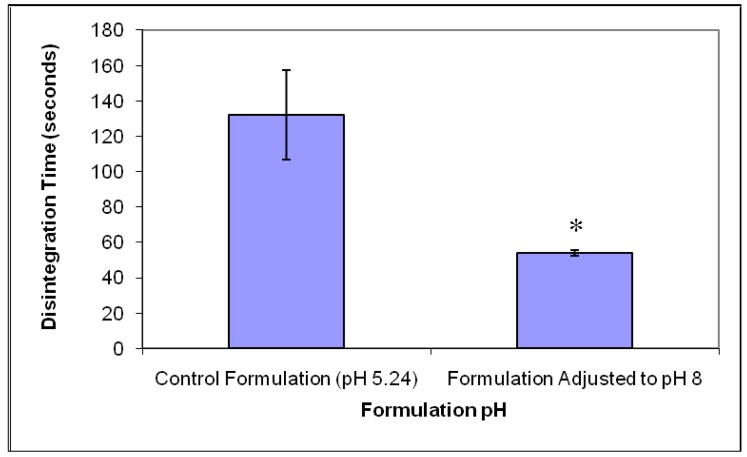
The effect of formulation pH on the disintegration time of the freeze-dried tablets (mean ± S.E., n = 3) (* statistically different).

Analysis of the disintegration time of the formulation adjusted to pH 8, as shown in Figure 4, indicated a mean disintegration time of 54 ± 1.7 s, whereas the formulation prepared at pH 5 exhibited a mean disintegration time of 132 ± 25.4 s. Adjustment of pH resulted in the reduction of disintegration time by over a half (*p* < 0.05), as shown in Figure 4. This observation can be attributed to two factors. Firstly, porosity and SEM studies revealed that the tablets prepared at pH 8 had higher porosity and thinner pore walls which could result in an increase in water uptake and subsequent better wetting/dispersibility [26]. Secondly, previously published reports have shown that aqueous solubility of gelatin is influenced by variations in pH with values above the isoelectric point of the material exhibiting an increase in its solubility [11,12].

### 3.2. Influence of Ionic Strength

To investigate the influence of ionic strength, various ratios of sodium chloride were incorporated in the formulation with gelatin and mannitol. The results revealed that sodium chloride had a concentration dependant influence. Formulations comprising of a 1:40 molar ratio of gelatin:sodium chloride resulted in a collapse of the final product with no tablet formation. Lower molar ratios (1:5, 1:10, 1:20 and 1:30) produced intact tablets and were characterized further for mechanical as well as thermal properties.

#### 3.2.1. Glass Transition and Tablet Mechanical Properties Analysis

DSC analyses of formulations with 1:5–1:30 molar ratios of gelatin:sodium chloride, as shown in Table 2, indicated mean onset T_g_ values comparable to the control formulation. The mean onset *T*_g_ values were −29.3 ± 0.1 °C, −30.5 ± 0.1 °C, −31.2 ± 0.2 °C and −31.7 ± 0.2°C, for 1:5, 1:10, 1:20 and 1:30 ratios, respectively, whilst the control formulation exhibited a mean onset *T*_g_ value of −29.8 ± 0.5 °C. Statistical analysis of the results indicated that the formulations consisting of gelatin:sodium chloride in molar ratios of 1:20 and 1:30, exhibited mean onset *T*_g_ values which were significantly different (*p* < 0.01) from the control formulation.

These results are in coherence with previous research investigating the effect of cations and anions of various electrolytes on the glass transition temperature of frozen solutions of excipients commonly used in freeze-drying, resulting in a decrease in glass transition temperature upon increasing ion concentration [27]. Formulations consisting of 1:20 and 1:30 gelatin:sodium chloride molar ratios, did exhibit significantly different mean onset T_g_ values relative to the control formulation (*p* < 0.01). However, as the difference was only around 2 °C, the structural collapse/shrinkage seen with the tablets of the formulation consisting of a gelatin:sodium chloride molar ratio of 1:40 was considered a physical rather than a thermal stability issue.

The inclusion of sodium chloride in the formulations with gelatin:sodium chloride molar ratios of 1:5–1:30 did not result in a significant increase in tablet hardness when compared to the control (*p* > 0.05), as shown in Table 3. Formulations with gelatin:sodium chloride molar ratios of 1:5, 1:10, 1:20 and 1:30, exhibited hardness values of 63.6 ± 5.6 N, 63.2 ± 8.0 N, 70.1 ± 2.1 N and 67.8 ± 8.3 N, respectively, whilst the control formulation had a mean value of 52.4 ± 8.8 N.

Analysis of the fracturability of the tablets of formulations with gelatin:sodium chloride molar ratios of 1:5–1:30 indicated that there was no statistical difference in the fracturability values of the various ratios of sodium chloride when compared to the control.

#### 3.2.2. Tablet Porosity, SEM and Disintegration Time Analysis

The inclusion of sodium chloride in the formulations resulted in differences in porosity of the tablets. Statistical analysis of the results indicated that the formulations consisting of gelatin:sodium chloride molar ratios of 1:5, 1:20 and 1:30, showed tablet porosities which were statistically significant (*p* < 0.01), when compared to the tablet porosity of the control formulation. There appeared to be a general trend that increasing the molar ratio of gelatin:sodium chloride from 1:5 to 1:20, produced a general increase in tablet porosity (1:5, 1:10 and 1:20, exhibited tablet porosity values of 88.70 ± 0.08%, 87.70 ± 0.08% and 89.50 ± 0.10%, respectively). The differences in porosity upon inclusion of sodium chloride can potentially be attributed to the differences in swelling behavior of gelatin in the presence of monovalent ions. Previous research has highlighted that the presence of sodium chloride had a bearing on the cross-linking of gelatin strands [28]. It is possible that inclusion of sodium chloride reduced the cross-linking during gelation which subsequently influenced tablet porosity.

**Figure 5.**
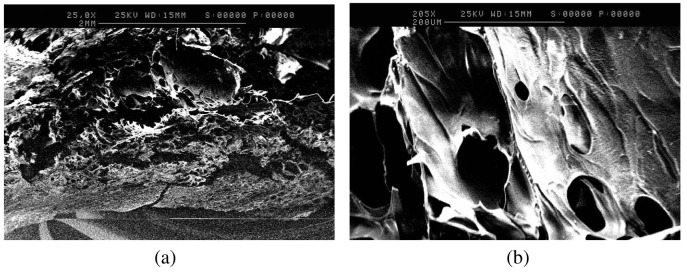
(**a**) SEM image of the tablet matrix of the 1:30 gelatin:soluble salt molar ratio formulation. Vertical Sample. Low Magnification, ×25; (**b**) SEM image of the tablet matrix of the 1:30 gelatin:soluble salt molar ratio formulation. Vertical Sample. High Magnification, ×205.

These results were further confirmed by SEM. SEM micrographs showed that the inclusion of sodium chloride in the formulations produced porous structures which generally exhibited the formation of larger pores and thinner pore walls (Figure 5a,b), compared to the porous structure of the control formulation (Figure 3a,b).

Horizontal sections of the tablets with gelatin:sodium chloride molar ratios of 1:10, 1:20 and 1:30, revealed average pore diameters of 100, 210 and 120 μm, respectively. Interestingly, tablets with a 1:20 gelatin:sodium chloride molar ratio, exhibited the greatest tablet porosity and shortest disintegration time of the four gelatin-soluble salt formulations. SEM analysis of the tablets revealed that increasing gelatin:soluble salt molar ratio appeared to increase disruption/damage to the porous matrix structures. The formulation with gelatin:sodium chloride molar ratio of 1:30, in particular, exhibited structural instability (as it appeared that pores had collapsed, forming cavities in the matrix) as shown in Figure 5a,b. This was likely due to the higher gelatin:soluble salt molar ratio, which appeared to weaken the structure, which led to the collapse of pores.

Analysis of the tablet disintegration times of formulations with gelatin:sodium chloride molar ratios of 1:5–1:30, showed that the inclusion of sodium chloride in the formulations with gelatin:sodium chloride molar ratios of 1:10 (126.7 ± 4.0 s) and 1:20 (102.3 ± 10.1 s), did not produce a significant reduction in disintegration time compared to the control formulation (132.0 ± 25.6 s mean disintegration time) (*p* > 0.05). The formulation with a gelatin:sodium chloride molar ratio of 1:5 produced a disintegration time (134.7 ± 12.0 s) comparable to the control formulation.

### 3.3. Milling Study

Ball milling has several pharmaceutical applications, which rely on a number of milling factors/parameters, such as; milling time, number of milling balls and milling jar volume, to fulfill their applications. These milling factors/parameters have a large range of operation, for e.g. milling time can range from a few minutes to several hours, thus making the possible number of milled sample formulations very large. Hence, factorial-experimental design software was used in order to propose a more suitable/manageable number of formulations (as shown in Table 1), which underwent various milling conditions based on three parameters; milling time, rotation speed and ball:powder weight ratio.

#### 3.3.1. Wettability Analysis

**Figure 6.**
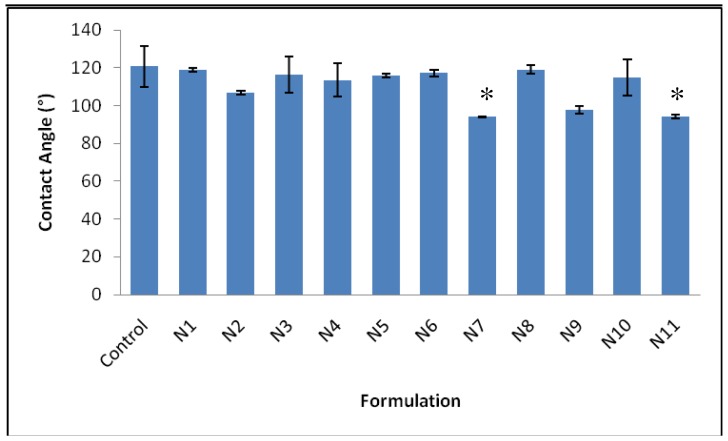
Analysis of the wettability of the milled formulations, expressed through their determined contact angle (mean ± S.E., n = 3) (* statistically different).

The wettability analysis results are shown in Figure 6. Formulations N7 (milling conditions; milling time of 15 minutes, at a rotation speed of 400 rpm, with a ball:powder weight ratio of 15) and N11 (milling conditions; milling time of 37.5 minutes, at a rotation speed of 250 rpm, with a ball:powder weight ratio of 10) showed the lowest contact angles, 94.0 ± 0.3° and 94.1 ± 1.0° respectively, and thus the greatest wettability (*p* < 0.05), whilst the control formulation exhibited a contact angle of 120.7 ± 11.0°. Milling is regularly used for the reduction of particle size [16], the observed improvement in wettability as a result of ball milling, can be attributed to a reduction in particle size and subsequent increase in surface area of the formulations [29].

#### 3.3.2. Powder Porosity Analysis

A significant variation in powder porosity between the formulations was recorded, as some of the mixtures exhibited greater porosity than the control (non-milled, 62.1 ± 0.3%), whilst others showed lower porosity. Formulation N8 showed the greatest porosity of 69.55 ± 0.1%, whilst formulation N6 produced the lowest value of 49.19 ± 0.2%. The porosity of the studied formulations is associated with their bulk density [30]. Therefore, as porosity varies between the formulations, so do their bulk density, which is related to the way in which the particles of the formulations are packed, during sample porosity analysis [30]. As milling is associated with particle size reduction, the various milling conditions are expected to produce a range of differing particle sizes. It is therefore expected that the way these particles pack during porosity analysis varies greatly, which results in differences in inter-particulate void spaces and subsequent variation in porosity between the formulations. All eleven of the formulations exhibited statistical significance, which indicates that ball milling has a significant effect on the porosity of the powders. Formulation N10 had a *p* value of <0.05, whilst all the other formulations had a *p* value of <0.01.

#### 3.3.3. Glass Transition and Tablet Mechanical Properties Analysis

DSC analysis of freeze-dried products is essential in order to fully appreciate and understand critical formulation properties such as the collapse temperature of the formulation [31]. The macroscopic collapse temperature of a formulation is defined as the temperature above which the freeze-dried product loses macroscopic structure and collapses during freeze-drying [32]. The macroscopic collapse temperature is closely related to the glass transition temperature of the formulation in its frozen state [33]. Therefore, in order to produce an acceptable freeze-dried product, it is always required to freeze dry a formulation at a temperature lower than the macroscopic collapse temperature [34,35].

Six of the eleven formulations exhibited glass transition temperatures which were considered not statistically significant (*p* > 0.05), when compared to the mean glass transition temperature of the control (non-milled) formulation. It can therefore be concluded that ball milling does not adversely affect the physical stability of the formulations in their frozen state or induce a plasticization effect, as comparable glass transition temperatures were observed.

All eleven of the formulations produced tablet hardness values which were not statistically significant (*p* > 0.05), when compared to the value of the control (non-milled) formulation (62.2 ± 3.8 N). Formulation N5 had the tablet hardness value of 55.0 ± 5.0 N, whilst formulation N10 produced the tablets which had hardness of 65.3 ± 1.0 N.

Similar results were obtained for fracturability analysis as no significant differences were observed when compared to the non-milled control formulation.

#### 3.3.4. Tablet Porosity, SEM and Disintegration Time Analysis

Tablet porosity is a critical property of ODTs, as highly porous tablets allow the rapid penetration of saliva into the tablet, which results in rapid oral disintegration. Tablet porosity significantly impacts the initial wetting and dispersion of active pharmaceutical ingredients [26]. It is therefore advisable to make tablets as porous as possible in order to achieve rapid disintegration. However, it is important to note that the physical/mechanical properties of the tablets such as hardness, should be maintained [26]. In general it is considered that increasing tablet porosity leads to an increase in water uptake and subsequent better wetting/dispersibility of active pharmaceutical ingredients [26], and consequently tablets exhibit shorter disintegration times.

The control formulation had porosity of 93.4 ± 0.4%. Formulation N6 produced tablets with the highest porosity of 94.4 ± 0.3%, whilst formulation N5 resulted in tablets with the lowest porosity of 92.9 ± 0.1%.

All studied formulations exhibited tablet porosities which were not statistically significant (*p* > 0.05), when compared to the tablet porosity of the control formulation.

These data were further confirmed with the SEM analysis as no microscopic differences were observed between milled and non-milled formulations.

The disintegration time was slightly different between the formulations. The control (non-milled) had a mean disintegration time of 23 ± 1 s. Tablets from formulation N5 exhibited the greatest disintegration time of 28 ± 2 s, whilst formulations N2, N10 and N11 all had the shortest disintegration time of 21 s.

#### 3.3.5. Dissolution Study Analysis

**Figure 7.**
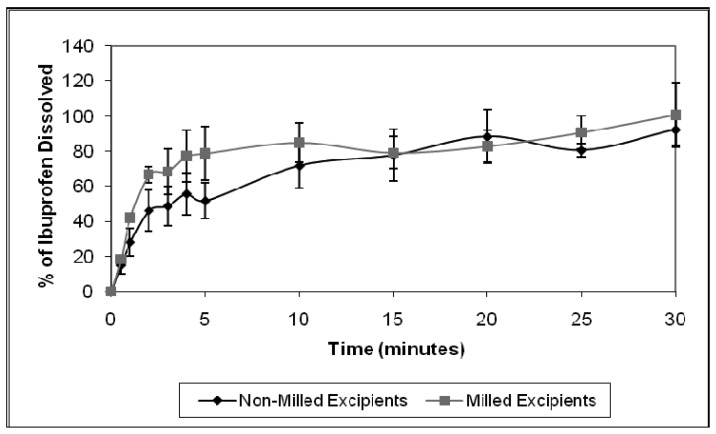
Dissolution profile of ibuprofen from lyophilized ODTs, prepared from non-milled and milled tablet excipients (mean ± S.E., n = 3).

Figure 7 illustrates the mean dissolution results for ibuprofen tablets, prepared from non-milled and milled tablet excipients. Although the time required for 80% ibuprofen dissolution from the tablets prepared from non-milled and milled materials, were 20 and 10 minutes, respectively, statistical analysis of the results indicated that there was no significant difference (*p* > 0.05) in the dissolution behavior of ibuprofen from the two tablet formulations. The results have therefore shown that milling the excipients did not influence ibuprofen dissolution from the lyophilized ODTs.

Tablet porosity and disintegration time are both critical properties in determining active pharmaceutical ingredient dissolution, as tablet porosity in particular, significantly impacts the initial wetting and dispersion of active pharmaceutical ingredients [26]. Initial results from this milling study, indicated that ball milling did not significantly affect tablet porosity and tablet disintegration time, when compared with tablets prepared from non-milled excipients. Therefore, comparable dissolution profiles of ibuprofen from tablets prepared from non-milled and milled materials was expected.

## 4. Conclusions

The study has shown that process parameters such as pH adjustment can have a significant influence on the disintegration time of gelatin based orally disintegrating tablets. The reduction in disintegration time did not compromise tablet hardness, which is a key parameter to measure ODT performance. The reduction in disintegration time can be attributed to an increase in tablet porosity, which allows the more rapid penetration of saliva or disintegrating medium into the tablet matrix, and an increase in gelatin solubility. The inclusion of sodium chloride in the formulations, to modify the ionic strength of the formulations, had an effect on tablet porosity and the glass transition of the formulations. However, inclusion of sodium ions is concentration dependent, with tablets comprising of higher salt concentration resulting in structural collapse/shrinkage. The study has also shown that ball milling influences formulation characteristics, such as powder porosity, and improves powder wettability.

## Figures and Tables

**Table 1.** Ball milling conditions of the various formulations.

**Formulation**	**Milling Time (minutes)**	**Rotation Speed (rpm)**	**Ball: Powder Weight Ratio**
N1	15	100	5
N2	60	100	5
N3	15	400	5
N4	60	400	5
N5	15	100	15
N6	60	100	15
N7	15	400	15
N8	60	400	15
N9	37.5	250	10
N10	37.5	250	10
N11	37.5	250	10

**Table 2.** Glass transition data of the formulations consisting of various ratios of gelatin:sodium chloride.

**Formulation**	**Mean Onset *T*_g_ (°C)**	**Standard Error**
Control	−29.8	0.5
1:5	−29.3	0.1
1:10	−30.5	0.1
1:20	−31.2	0.2
1:30	−31.7	0.2

**Table 3.** Tablet hardness data of the formulations consisting of various ratios of gelatin:sodium chloride.

**Formulation**	**Mean Tablet Hardness (N)**	**Standard Error**
Control	52.4	8.8
1:5	63.6	5.6
1:10	63.2	8.0
1:20	70.1	2.1
1:30	67.8	8.3
